# Efficient photocatalytic hydrogen evolution on single-crystalline metal selenide particles with suitable cocatalysts†

**DOI:** 10.1039/d0sc01167c

**Published:** 2020-04-01

**Authors:** Shanshan Chen, Junie Jhon M. Vequizo, Takashi Hisatomi, Mamiko Nakabayashi, Lihua Lin, Zheng Wang, Akira Yamakata, Naoya Shibata, Tsuyoshi Takata, Taro Yamada, Kazunari Domen

**Affiliations:** Research Initiative for Supra-Materials, Interdisciplinary Cluster for Cutting Edge Research, Shinshu University 4-17-1 Wakasato, Nagano-shi Nagano 380-8553 Japan domen@chemsys.t.u-tokyo.ac.jp; Institute of Engineering Innovation, The University of Tokyo Tokyo 113-8656 Japan; Graduate School of Engineering, Toyota Technological Institute 2-12-1 Hisakata, Tempaku Nagoya 468-8511 Japan; Office of University Professors, The University of Tokyo 7-3-1 Hongo, Bunkyo-ku Tokyo 113-86556 Japan

## Abstract

It is important to improve the apparent quantum yields (AQYs) of narrow bandgap photocatalysts to achieve efficient H_2_ production. The present work demonstrates a particulate solid solution of zinc selenide and copper gallium selenide (denoted as ZnSe:CGSe) that evolves H_2_ efficiently and is responsive to visible light up to 725 nm. This material was synthesized using a flux-assisted method and was found to comprise single-crystalline tetrahedral particles. The coloading of Ni and Rh, Pt, Pd or Ru as cocatalysts further improved the photocatalytic H_2_ evolution rate over this photocatalyst. With the optimal coloading of a Ni–Ru composite cocatalyst, an AQY of 13.7% was obtained at 420 nm during a sacrificial H_2_ evolution reaction, representing the highest value yet reported for a photocatalyst with an absorption edge longer than 700 nm. The present study demonstrates that the preparation of single-crystalline particles and the rational assembly of composite cocatalysts are effective strategies that allow the efficient utilization of long wavelengths by metal selenide photocatalysts for solar fuel production.

## Abstract 2

Coloading of a Ni–Ru composite cocatalyst on a 700 nm-class single-crystalline particulate selenide photocatalyst improves its hydrogen evolution activity.

## Graphical abstract

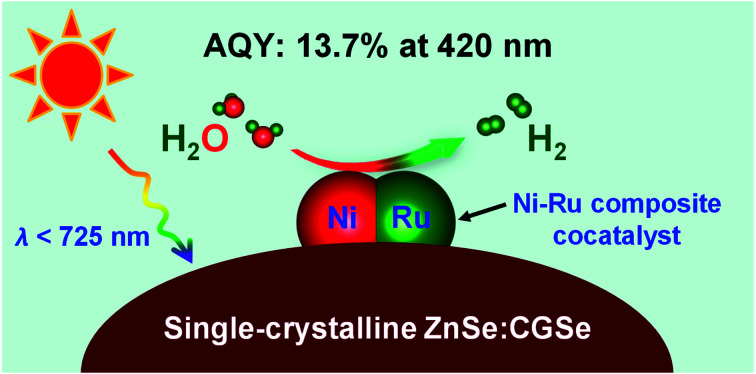

## Introduction

Metal selenides are narrow bandgap semiconductors with tunable electronic and band structures and thus have been widely applied, such as in solar cells, light-emitting diodes, photoelectrochemical devices and biomedical diagnostics.^1–4^ Sunlight-driven water splitting using particulate photocatalysts is of vital importance in consideration that the produced hydrogen is an environmentally friendly and sustainable alternative to fossil fuels.^5–9^ A prerequisite for efficient solar energy conversion is the development of photocatalytic systems that can utilize the wide wavelength range of the solar spectrum.^10^ Metal selenide semiconductors are promising photocatalytic materials because they have narrow bandgaps and tunable band structures.^11,12^

Selenide semiconductors have been used primarily in the sacrificial H_2_ evolution reaction because the valence band maximums (VBMs) of most selenides are more negative than the water oxidation potential, such that photogenerated holes preferentially oxidize the selenides themselves (*via* photocorrosion) rather than water.^13^ However, the application of metal selenides in non-sacrificial photocatalytic pure water splitting was recently demonstrated based on the construction of an effective Z-scheme system. In this system, metal selenides and CoO_*x*_/BiVO_4_ were used as hydrogen evolution photocatalysts (HEPs) and an oxygen evolution photocatalyst (OEP), respectively.^11,12^ The selenides comprised solid solutions of zinc selenide (ZnSe) and copper gallium selenide (CGSe) (denoted as ZnSe:CGSe) prepared using a solid-state reaction.^14^ However, the apparent quantum yield (AQY) obtained from such systems was only about 0.5% at 420 nm during overall water splitting, which was much lower than that of a SrTiO_3_:La,Rh/Au/BiVO_4_:Mo system that incorporates a similar electron mediator and an OEP.^11,15^ It is thought that the rate-determining step in selenide-based photocatalytic systems is likely associated with the HEP part, and so it is necessary to improve the activity of metal selenides during photocatalytic H_2_ evolution so as to increase the net solar water splitting efficiency.

Water splitting photocatalysts are typically composed of a light-harvesting semiconductor and one or more cocatalysts.^16,17^ The semiconductor is primarily responsible for light absorption and charge transfer, while catalytic reactions occur mainly on the surfaces of the cocatalysts.^18^ Therefore, both the selenide semiconductors and cocatalysts need to be finely tuned to improve the cumulative efficiency of the elementary steps (light absorption, charge transfer and catalytic conversion) so as to produce an efficient metal selenide photocatalyst. In the present work, a flux-assisted preparation method was developed to synthesize single-crystalline ZnSe:CGSe particles.^19^ This material had a tetrahedral particle morphology and exhibited a long absorption edge of 725 nm. Moreover, the coloading of Ni and various noble metals (Rh, Pt, Pd or Ru) as cocatalysts was found to enhance photocatalytic H_2_ evolution over this material. The AQY obtained using this system reached 13.7% at 420 nm, which is approximately 27 times higher than the value previously reported for this material (0.5% at 420 nm) and, in fact, represents the highest AQY yet obtained using a 700 nm-class photocatalyst.^12^ The strategies demonstrated herein are believed to have applications in other photocatalytic systems so as to achieve efficient solar fuel production.

## Results and discussion

**Fig. 1:**
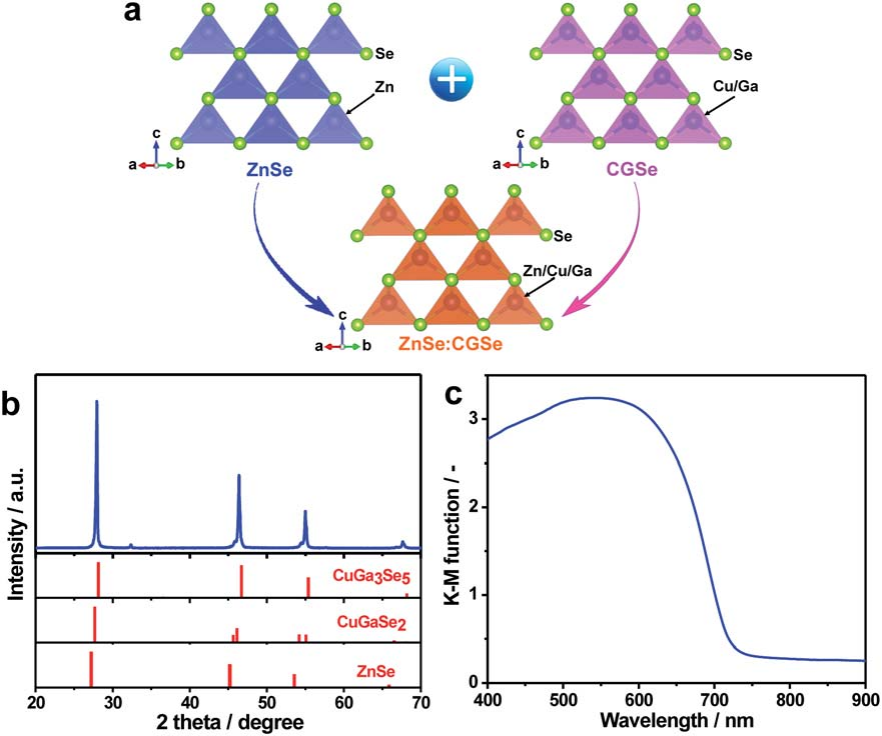
Structural analyses of ZnSe:CGSe: (a) a schematic showing the crystal structure of a solid solution of ZnSe and CGSe, (b) the XRD pattern and (c) the UV-vis DRS obtained from the prepared ZnSe:CGSe sample.

ZnSe:CGSe solid solution was synthesized using a flux-assisted method in sealed evacuated quartz tubes, as described in the ESI.† The Ga/Cu and Zn/(Zn + Cu) molar ratios were fixed at 2.0 and 0.5, respectively, because a ZnSe:CGSe solid solution with this composition has previously demonstrated high Z-scheme overall water splitting activity when used as a HEP.^11^Fig. 1a presents a schematic showing the crystal structures of the constituent solid solutions. The ZnSe:CGSe solid solution is a quaternary complex selenide, whose crystal structure is similar to a ZnSe zinc blende structure and a CGSe chalcopyrite structure.^20^ Zn^2+^ (with an ionic radius of 74 pm) occupies both Cu^+^ (74 pm) and Ga^3+^ (61 pm) sites during the formation of the solid solution, and each metal cation is tetrahedrally coordinated to four Se^2−^ ions. In the X-ray diffraction (XRD) pattern obtained from this material (Fig. 1b), the main diffraction peaks were located between those for standard ZnSe (ICSD #162755) and CuGa_3_Se_5_ (ICSD #181418) specimens, demonstrating the successful formation of a solid solution. More specifically, the as-prepared ZnSe:CGSe (molar ratio of Ga/Cu was 2.0) specimen was composed of the transition phase of CuGaSe_2_ and CuGa_3_Se_5_.^11,14^ Analysis using the UV-vis diffuse reflectance spectrum (DRS; Fig. 1c) showed that the present ZnSe:CGSe specimen was a narrow band gap semiconductor with an absorption edge of 725 nm, similar to ZnSe:CGSe prepared using the traditional solid-state reaction method.^11^

**Fig. 2:**
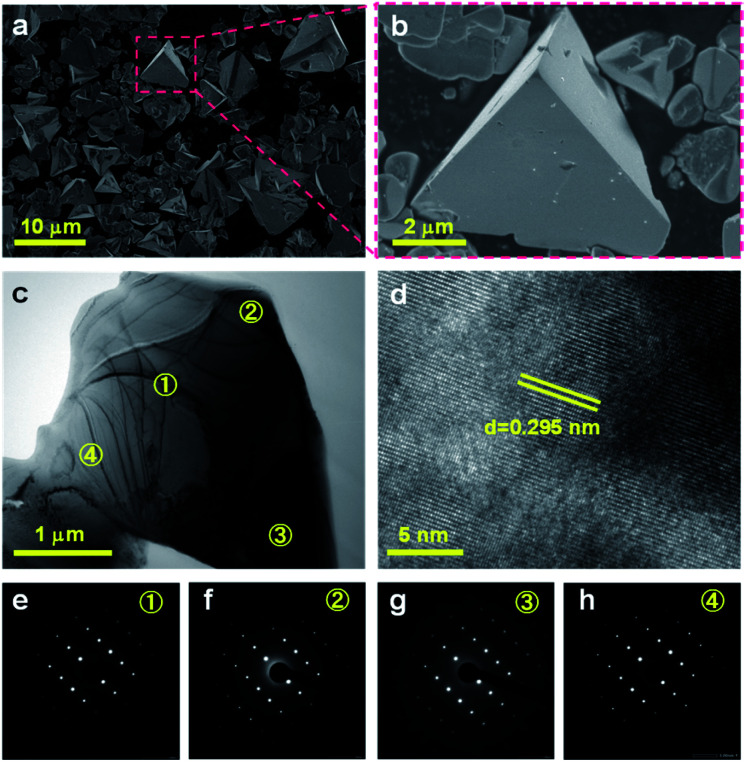
(a, b) SEM images of the as-prepared ZnSe:CGSe powder, (c, d) cross-sectional views and (e–h) SAED patterns of a single ZnSe:CGSe particle. The numbers in e–h correspond to the labelled positions in c.

Scanning electron microscopy (SEM) images (Fig. 2a and b) indicated that the selenide solid solution was primarily composed of tetrahedral particles whose sizes ranged from several to 10 μm. In order to further examine the microstructure of the ZnSe:CGSe particles, Ar ion milling was used to prepare cross-sections of particles. The clear lattice fringes observed in the cross-sectional view of a single ZnSe:CGSe particle (Fig. 2c and d) demonstrated the high degree of crystallinity of the material. Selected area electron diffraction (SAED) analyses were performed at different locations (as indicated in Fig. 2c) to assess the lattice orientation. As shown in Fig. 2e–h, identical facet orientations were observed at these different positions. Therefore, it can be concluded that our prepared ZnSe:CGSe material comprised single-crystalline particles having tetrahedral morphologies.

**Fig. 3:**
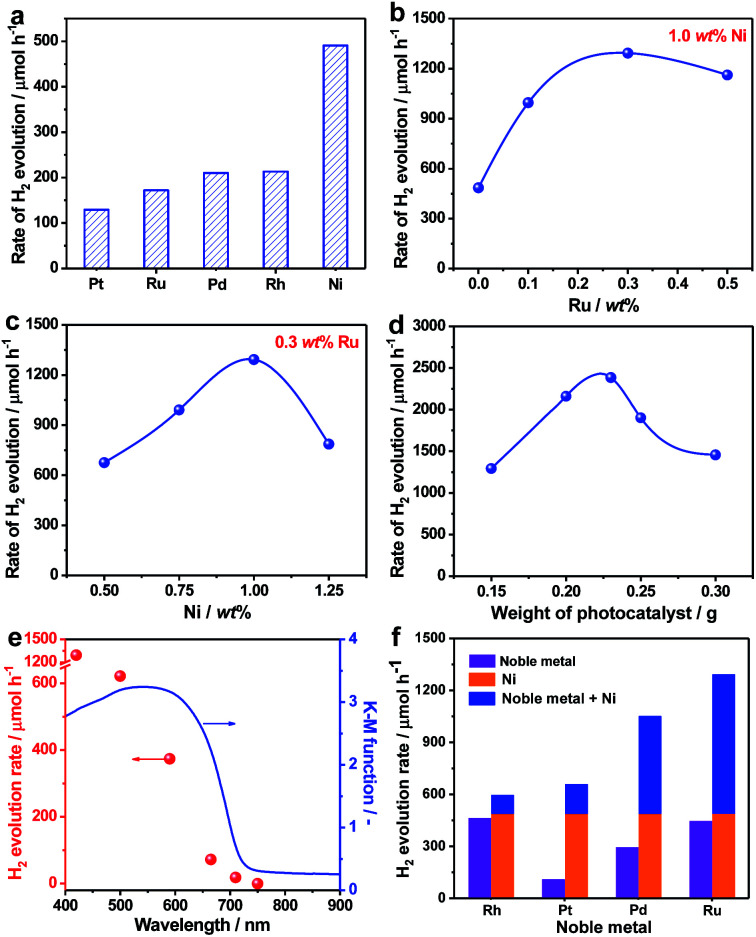
Photocatalytic H_2_ performance over ZnSe:CGSe-based photocatalysts: (a) H_2_ evolution rates over ZnSe:CGSe photocatalysts modified with different cocatalysts at 1.0 wt% in each case. Optimization of the Ni and Ru loading amounts with regard to photocatalytic H_2_ production: (b) the effects of the Ru loading with 1.0 wt% Ni, (c) the effects of the Ni loading with 0.3 wt% Ru. (d) Effect of the amount of the 1.0 wt% Ni–0.3 wt% Ru/ZnSe:CGSe photocatalyst on the photocatalytic H_2_ evolution rate. (e) The dependence of the H_2_ evolution rate of the 1.0 wt% Ni–0.3 wt% Ru/ZnSe:CGSe photocatalyst on the cutoff wavelength of incident light (red points) and the UV-vis DRS of ZnSe:CGSe (blue line). (f) H_2_ evolution rates over ZnSe:CGSe photocatalysts loaded with various cocatalysts under visible light irradiation. Experimental conditions: for (a–c, e and f): 0.15 g photocatalyst (1.0 wt% and 0.3 wt% were fixed to the Ni and noble metal, respectively, if not indicated), 0.1 mol L^−1^ Na_2_S and 0.1 mol L^−1^ Na_2_SO_3_, 150 mL H_2_O, 300 W Xe light, *λ* > 420 nm; for (d) 0.5 mol L^−1^ Na_2_S and 0.5 mol L^−1^ Na_2_SO_3_, 150 mL H_2_O, 300 W Xe light, *λ* > 420 nm.

Due to their unique single crystalline nature, it was anticipated that the as-prepared ZnSe:CGSe particles could act as a good platform for an efficient photocatalytic process. The H_2_ evolution reaction was thus investigated based on the aforementioned analysis in the part of introduction. In initial trials, several typical cocatalysts (Pt, Ru, Pd, Rh and Ni) were introduced into the material using an *in situ* precipitation method (see details in the Experimental section in the ESI†) at a loading amount of 1.0 wt% in each case. The highest photocatalytic H_2_ evolution rate of 491 μmol h^−1^ was obtained with Ni (Fig. 3a). It is noteworthy that, at the same Ru loading, the H_2_ evolution rate obtained from the single-crystalline ZnSe:CGSe particles (172 μmol h^−1^) was 7.2 times higher than that of ZnSe:CGSe having the same composition (24 μmol h^−1^) but prepared by the traditional solid-state reaction, demonstrating the advantage of the flux-assisted method.^12^ The relationship between the H_2_ evolution rate and Ni loading produced a volcano-shaped plot that peaked at a loading of 1.0 wt% (Fig. S1†). The photocatalytic H_2_ production was further improved by coloading of both Ni and Ru. As shown in Fig. 3b and c, a volcano-shaped relationship was evident when plotting the H_2_ evolution rate against the proportion of either Ru or Ni. The corresponding time courses of H_2_ evolution are presented in Fig. S2.† From these data, it was determined that the optimal coloading proportions of Ni and Ru were 1.0 wt% and 0.3 wt%, respectively, leading to the highest H_2_ evolution rate of 1293 μmol h^−1^. Interestingly, this rate exceeded the sum of the rates obtained using 1.0 wt% Ni/ZnSe:CGSe (491 μmol h^−1^) and 0.3 wt% Ru/ZnSe:CGSe (445 μmol h^−1^). Control experiments showed that no appreciable amount of H_2_ was produced when Ni, Ru or a Ni–Ru combination was used as the photocatalyst, indicating that these materials were inactive for photocatalytic H_2_ production. From these results, it can be concluded that there was a synergistic effect between Ni and Ru species when they were coloaded as cocatalysts on ZnSe:CGSe for photocatalytic H_2_ evolution.

In an attempt to maximize the H_2_ evolution rate of the newly developed 1.0 wt% Ni–0.3 wt% Ru/ZnSe:CGSe photocatalyst, the operational conditions were optimized. Fig. 3d and S3† summarize the H_2_ evolution rates and the corresponding time courses of evolved H_2_ obtained using the 1.0 wt% Ni–0.3 wt% Ru/ZnSe:CGSe photocatalyst in varying amounts. In these reactions, the sacrificial reagents at the greater concentrations were used to weaken the effect of the concentration decrease during the reaction. With an increase in the photocatalyst quantity, the H_2_ evolution activity was enhanced to a maximum rate of 2390 μmol h^−1^ at a catalyst mass of 0.23 g. With a further increase in the amount of photocatalyst, the activity decreased, likely due to the reduced light-harvesting efficiency resulting from light scattering. An AQY of 13.7% was achieved at 420 nm in conjunction with the optimized reaction conditions, representing the highest value yet reported for a photocatalyst with an absorption edge longer than 700 nm (Table S1†). In comparison, the AQY was only 0.5% at 420 nm when this same selenide solid solution was prepared using the traditional solid-state method in our previous work.^12^ Thus, the AQY was remarkably enhanced by employing single-crystalline particles and coloading the Ni–Ru composite cocatalyst. The time courses of the evolved H_2_ amounts in Fig. S3† demonstrate that the H_2_ evolution rate slightly decreased over time when these photocatalysts were used. This effect may have been due partly to the chemical corrosion of the selenides in the reaction solution, which contained high concentrations of Na_2_S and Na_2_SO_3_. As shown in Fig. S4,† Se^2−^ ions were eluted from the photocatalyst into the reaction solution, likely owing to the exchange of S^2−^ for Se^2−^ ions.^21^ This anion exchange process evidently degraded the H_2_ evolution rate of the selenide photocatalyst (Fig. S5†). However, this process did not occur when the ZnSe:CGSe photocatalyst was assembled to fabricate a photocatalytic Z-scheme water splitting system with pure water as the reaction solution, as demonstrated in our previous work.^11,12^

The dependence of the H_2_ evolution rate on the cutoff wavelength of incident light is presented in Fig. 3e. The onset of H_2_ evolution was found to be consistent with the onset of absorption as demonstrated by the UV-vis DRS data, confirming that the photoreaction occurred *via* bandgap transitions. It should be noted that incident light with wavelengths longer than 710 nm could still be utilized to drive the H_2_ evolution reaction, showing the ability of the ZnSe:CGSe to function at especially long wavelengths. In addition to the combination of Ni and Ru, combinations of Ni and Rh, Pt or Pd also provided similar enhancements of photocatalytic H_2_ production over the ZnSe:CGSe (Fig. 3f), using Ni and noble metal loadings of 1.0 wt% and 0.3 wt%, respectively. Among these composite cocatalysts, the Ni–Ru cocatalyst produced the highest H_2_ evolution rate. Therefore, this combination was used in subsequent trials to obtain insights into its intriguing photocatalytic performance.

**Fig. 4:**
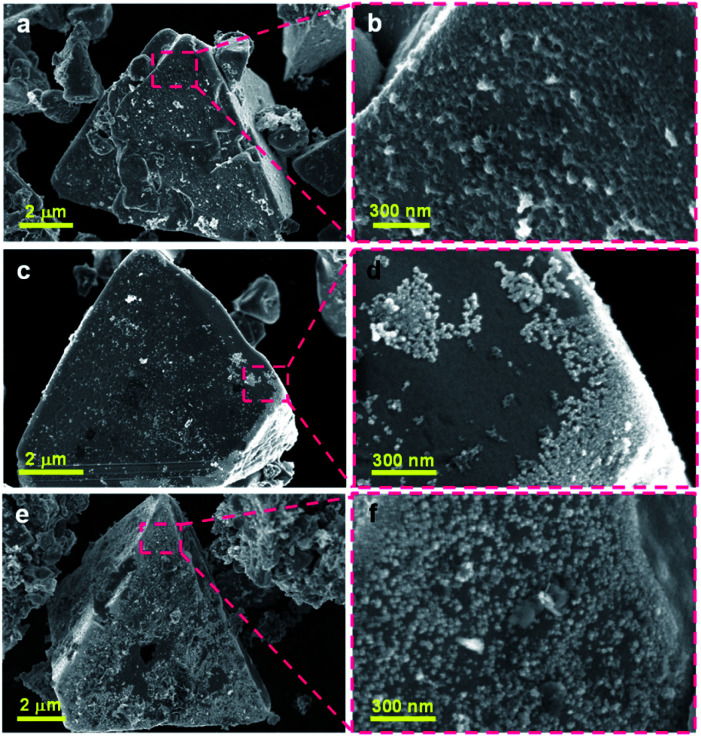
SEM images of the ZnSe:CGSe-based photocatalyst loaded with different cocatalysts: (a, b) 1.0 wt% Ni/ZnSe:CGSe, (c, d) 0.3 wt% Ru/ZnSe:CGSe and (e, f) 1.0 wt% Ni–0.3 wt% Ru/ZnSe:CGSe.

To compare the dispersion states of the loaded cocatalysts, three representative photocatalysts (1.0 wt% Ni/ZnSe:CGSe, 0.3 wt% Ru/ZnSe:CGSe and 1.0 wt% Ni–0.3 wt% Ru/ZnSe:CGSe) were characterized by SEM. As shown in Fig. 4a and b, the deposited Ni species formed a floc and evenly coated the surfaces of the ZnSe:CGSe particles. However, the Ru species evidently generated aggregates of nanoparticles (Fig. 4c and d), while, in the case of the Ni–Ru coloaded sample (Fig. 4e and f), both Ni and Ru species were dispersed evenly. These results suggest that the *in situ* formed Ni-based floc provided a platform that prevented the Ru-based nanoparticles from aggregating.^22^ Analyses by scanning transmission electron microscopy and energy-dispersive X-ray spectroscopy (STEM-EDS) suggested that the deposited Ru and Ni species were generally separate in the same cocatalyst particles, thus forming a composite (Fig. S6 and S7†), although it was difficult to ascertain the exact structure because of the limited resolution of this analytical method.

The chemical compositions of the loaded cocatalysts in these three samples were probed by X-ray photoelectron spectroscopy (XPS) and the Ni 2p_3/2_ spectra are provided in Fig. S8a.† The Ni 2p_3/2_ peaks generated by the Ni/ZnSe:CGSe were deconvoluted into four peaks that could be assigned to the main (853.1 eV) and satellite (858.9 eV) peaks from NiS and the main (855.9 eV) and satellite (861.7 eV) peaks from Ni^2+^ in Ni(OH)_2_.^23,24^ The presence of the sulfide constituent was demonstrated by the S 2p spectra shown in Fig. S8b.† Based on these data, the deposited Ni species under the current experimental conditions was evidently in the form of a Ni(OH)_2_/NiS composite. The Ni–Ru/ZnSe:CGSe generated a similar Ni 2p_3/2_ spectrum to that of the Ni/ZnSe:CGSe, but the corresponding binding energies were slightly shifted to lower values. The Ru 3d_5/2_ peak in Fig. S8c† confirms that Ru-containing species were successfully deposited on the Ru/ZnSe:CGSe and Ni–Ru/ZnSe:CGSe samples. Based on the S 2p spectrum of Ru/ZnSe:CGSe (Fig. S8b†), the deposited Ru species were present as Ru_2_S_3_.^25^ In addition, some amounts of metallic Ru or Ni species could also have been formed *via* reduction reactions with photogenerated electrons.^23,25,26^ Notably, the S 2p and Ni 2p_3/2_ binding energies in the spectra obtained from the coloaded specimen were shifted to higher and lower values, respectively, than those in the samples loaded solely with Ni or Ru. This result can possibly be ascribed to interactions between the deposited Ni and Ru species.^27^

**Fig. 5:**
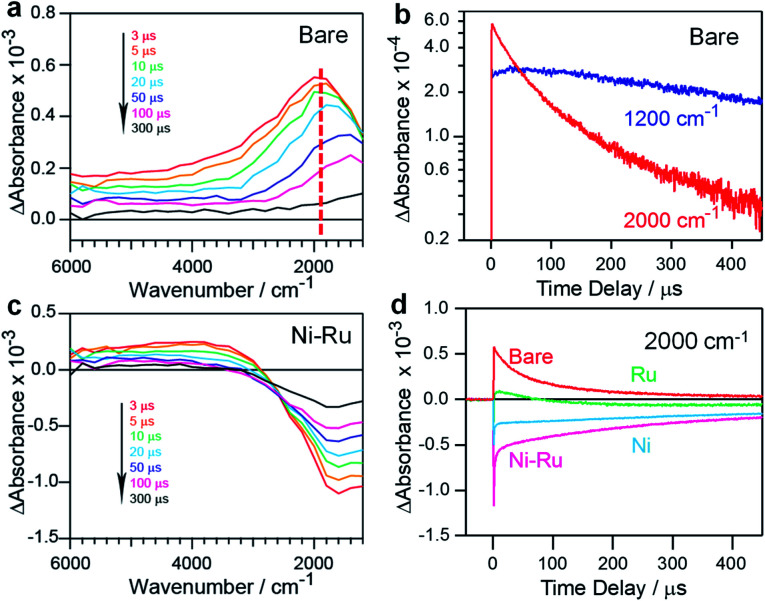
TA data obtained from various ZnSe:CGSe samples excited at 480 nm: (a) TA spectra of an unmodified ZnSe:CGSe sample, (b) kinetic profiles of electrons in an unmodified ZnSe:CGSe sample probed at 2000 and 1200 cm^−1^ in the μs region, (c) TA spectra of a 1.0 wt% Ni–0.3 wt% Ru/ZnSe:CGSe sample and (d) kinetic profiles of electrons in ZnSe:CGSe samples probed at 2000 cm^−1^ in the μs region. Here, unmodified ZnSe:CGSe, 1.0 wt% Ni/ZnSe:CGSe, 0.3 wt% Ru/ZnSe:CGSe and 1.0 wt% Ni–0.3 wt% Ru/ZnSe:CGSe are denoted as Bare, Ni, Ru and Ni–Ru.

To understand the effect of the coloaded cocatalysts on the dynamics of photogenerated charges in the ZnSe:CGSe, transient absorption (TA) spectroscopy was conducted. In these trials, the specimens were irradiated using 480 nm (2.58 eV) laser pulses that excited electrons from the valence band (VB) to the conduction band (CB) of the material. The mid-infrared TA spectra of bare ZnSe:CGSe were obtained over the range of 6000–1000 cm^−1^ (0.74–0.1 eV) to assess the dynamics of shallowly trapped electrons and free electrons excited in the CB.^28,29^ The spectra (Fig. 5a) showed increasing absorption intensity towards lower wavenumbers and a discernible absorption peak at approximately 1800 cm^−1^ (0.22 eV). These features are attributable to free electrons in the ZnSe:CGSe CB and electrons trapped at 0.22 eV below the CB, respectively. A slight red-shift of the peak at 1800 cm^−1^ was observed over time while acquiring the data. This effect is ascribed to the re-excitement of the photoexcited electrons in shallow traps to the CB through an Auger process in conjunction with electron–hole recombination, such that there was a lower population of the trapped electrons and a higher population of the CB electrons.^30,31^ To further examine this process, the changes in intensity at 1200 and 2000 cm^−1^ were compared, as these values reflect the transient kinetics of the free and trapped electrons, respectively (Fig. 5b). This comparison demonstrated that the intensity at 2000 cm^−1^ was higher than that at 1200 cm^−1^ at the initial stage after pumping, indicating that the trapped electrons were more populous. However, on longer timescales, the intensity at 1200 cm^−1^ was higher than that at 2000 cm^−1^, suggesting that the free electrons became dominant. Notably, the lifetime of the free electrons was longer than that of the trapped electrons.

In the case of Ni–Ru coloaded ZnSe:CGSe, the TA spectra showed positive absorption in the range of 6000–3000 cm^−1^ similar to that of the unmodified ZnSe:CGSe, together with relatively strong negative absorption in the region of 3000–1000 cm^−1^ (Fig. 5c). The positive absorption reflected electrons that may have been trapped at defects in the ZnSe:CGSe.^32,33^ The negative absorption was not attributed to IR emission because the IR contribution had already been subtracted from the spectra (as described in the ESI†). It has been reported that electron transfer from Cu_7_S_4_ to CdS induces negative absorption on the microsecond to millisecond time scales, and so the negative signal observed in the present work was similarly attributable to photoexcited electrons transferred from the ZnSe:CGSe to the cocatalysts.^34^ The recovery of the negative absorption would therefore represent the recombination of electrons transferred to the cocatalysts with holes in the ZnSe:CGSe. The intensity of the negative signal at 2000 cm^−1^ produced by the specimens was found to decrease in the order of Ni–Ru > Ni > Ru (Fig. 5d), suggesting that electron transfer was more efficient in the Ni–Ru loaded ZnSe:CGSe as compared to the sample containing solely Ru or Ni.

The above characterization results demonstrate that better dispersion and decreased agglomeration of the Ru-based nanoparticles were obtained by coloading with Ni. This provided maximum exposure and optimal distribution of the Ni–Ru cocatalyst, such that the photoexcited electrons were captured and utilized more efficiently. Consequently, enhanced electron transfer from the ZnSe:CGSe to the Ni–Ru composite cocatalyst was observed in the TA spectra. Notably, Ni(OH)_2_ has been shown to promote the dissociation of water molecules and the production of hydrogen intermediates that subsequently are adsorbed and recombine to produce molecular hydrogen on nearby metal or metal sulfide particles.^24,35–37^ This synergistic effect of Ni(OH)_2_ and NiS may have allowed the Ni cocatalyst to generate higher H_2_ evolution rates as compared to the noble metals, as shown in Fig. 3a. When it was further coupled with another metal sulfide, such as Ru_2_S_3_, the number of active sites was increased and the interaction with Ni(OH)_2_ was strengthened, leading to the maximal performance during this catalytic process (Fig. 3b). Therefore, it can be concluded that enhanced electron transfer efficiency from the selenide to the cocatalysts and accelerated H_2_ evolution kinetics on the Ni–Ru composite cocatalyst contributed to the remarkably improved H_2_ evolution rate. Nevertheless, it should be noted that the AQY value of slightly more than 10% obtained in the present work is still not overly high. This means that there remains a room to improve the quantum efficiency. One possible approach to enhancing the efficiency would be to improve the preparation procedure to make smaller (*i.e.* on the order of several hundred nanometers) and more homogeneous particle sizes of ZnSe:CGSe.

## Conclusions

ZnSe:CGSe solid solution and a series of Ni-based composite cocatalysts were developed towards efficient photocatalytic H_2_ production. ZnSe:CGSe powder with a single-crystalline nature and an absorption edge of 725 nm was prepared by a flux-assisted method. Based on this material, the coloading of Ni and several noble metals species was found to improve the photocatalytic H_2_ evolution rate. Among the various combinations investigated, a Ni–Ru composite cocatalyst produced the highest H_2_ evolution rate of 2390 μmol h^−1^ under visible light irradiation, with an AQY of 13.7% at 420 nm. This is the highest value obtained to date among 700 nm photocatalysts. The remarkably improved activity of this material is primarily attributed to the enhanced efficiency of electron transfer from the ZnSe:CGSe to the Ni–Ru cocatalyst together with accelerated H_2_ evolution kinetics. This work demonstrates that the development of particulate single-crystalline semiconductors and the engineering of specific composite cocatalysts are useful strategies for the design of efficient photocatalysts.

## Conflicts of interest

There are no conflicts to declare.

## Author contributions

K. D. led the research project. S. C. prepared the photocatalyst and conducted the H_2_ evolution reaction. J. V. and A. Y. carried out the TA measurement. M. N. and N. S. conducted transmission electron microscopy, STEM-EDS and SAED characterization. S. C. and Z. W carried out the measurement of AQY. T. Y. performed inductively coupled plasma-optical emission spectrometry (ICP-OES) analyses. S. C., T. H., J. V., L. L., M. N., A. Y., N. S., T. T. and K. D. discussed the results. S. C., T. H. and K. D. wrote the manuscript with contributions from the other co-authors. All authors reviewed the manuscript.

## Supplementary Material

SC-011-D0SC01167C-s001
