# Supplementary material for: Efficient photocatalytic hydrogen evolution on single-crystalline metal selenide particles with suitable cocatalysts
Source: Chem Sci. 2020 Apr 1;11(25):6436–41. doi: 10.1039/d0sc01167c (PMC8159321; doi:10.1039/d0sc01167c)
Supplement: SC-011-D0SC01167C-s001 [file SC-011-D0SC01167C-s001.pdf]

Electronic Supplementary Material (ESI) for Chemical Science.
This journal is © The Royal Society of Chemistry 2020

Efficient Photocatalytic Hydrogen Evolution on Single-Crystalline Metal Selenide Particles with Suitable Cocatalysts

Shanshan Chen,[1] Junie Jhon M. Vequizo,[1] Takashi Hisatomi,[1] Mamiko Nakabayashi,[2] Lihua Lin,[1] Zheng Wang,[1] Akira Yamakata,[3] Naoya Shibata,[2] Tsuyoshi Takata,[1] Taro Yamada,[4] and Kazunari Domen[1,4,*]

[1] Research Initiative for Supra-Materials, Interdisciplinary Cluster for Cutting Edge Research, Shinshu University, 4-17-1 Wakasato, Nagano-shi, Nagano 380-8553, Japan

[2] Institute of Engineering Innovation, The University of Tokyo, Tokyo 113-8656, Japan

[3] Graduate School of Engineering, Toyota Technological Institute, 2-12-1 Hisakata, Tempaku, Nagoya 468-8511, Japan

[4] Office of University Professors, The University of Tokyo, 7-3-1 Hongo, Bunkyo-ku, Tokyo 113-86556, Japan

*E-mail: domen@chemsys.t.u-tokyo.ac.jp

1. Experimental Section

1.1 Preparation of selenide semiconductors

Particulate solid solution of $(ZnSe)_{0.5}(CuGa_2Se_{3.5})_{0.5}$ (denoted as ZnSe:CGSe) was prepared by a flux-assisted method.[1] Typically, Cu_2Se (99.9%, High Purity Chemicals), Ga_2Se_3 (99.9%, High Purity Chemicals) and ZnSe (99.99%, High Purity Chemicals) reagents were ground with the Zn/(Zn+Cu) and Ga/Cu molar ratios of 0.5 and 2.0, respectively, in a glovebox for 20 min. The introduced flux of LiCl (99.0%, Wako Pure Chemical Industries, Ltd.) and KCl (99.9%, Wako Pure Chemical Industries, Ltd.) were dehydrated by heating at 200 °C for 2 h under vacuum prior to use. Then the dehydrated LiCl and KCl with a molar ratio of 3:2 were mixed with the pre-mixed precursor for another 20 min. The molar ratio of the precursor and flux was 1:5. The final mixture was sealed in a quartz ampoule and heated at 550 °C for 10 h. After the ampule was naturally cooled to room temperature, the calcined product was separated from the chloride flux by washing with distilled water for several times.

1.2 Deposition of cocatalysts and the photocatalytic H_2 evolution reaction

A closed gas circulation system with top irradiation from a 300 W Xe lamp (CX-04E INOTEX, Japan) equipped with a cut-off filter (λ > 420 nm, unless otherwise noted) was used for the photocatalytic H_2 evolution reaction. For each reaction, typically 0.15 g of selenide photocatalyst was suspended in 150 mL of an aqueous solution containing 0.1 mol L^{-1} Na_2S (97.0%, Wako Pure Chemical Industries, Ltd.) and 0.1 mol L^{-1} Na_2SO_3 (analytical grade, Kanto Chemical Co., Inc.). Cocatalysts were loaded by *in situ* precipitation method before the photocatalytic reaction.[2] For the bare selenide semiconductor suspended in the aforementioned aqueous solution containing the sacrificial reagents, a cocatalyst was loaded by adding a specific amount of $H_2PtCl_6{\cdot}6H_2O$ (98.5%, Wako Pure Chemical Industries, Ltd.), $RuCl_3{\cdot}3H_2O$ (99.9%, Wako Pure Chemical Industries, Ltd.), $PdCl_2$ (99.9%, Wako Pure Chemical Industries, Ltd), $RhCl_3{\cdot}3H_2O$ (95.0%, Wako Pure Chemical Industries, Ltd.), or $Ni(NO_3)_2{\cdot}6H_2O$ (99.9%, Wako Pure Chemical Industries, Ltd) to the photocatalyst suspension. It is expected

that the metal cations will react with S^{2-} to form metal sulfides, which will be adsorbed by the selenide material. For the procedure of cocatalysts coloading, two precursors including $Ni(NO_3)_2{\cdot}6H_2O$ were added simultaneously to the suspension before irradiation. Herein, the loading amount of the cocatalyst is indicated by the amount of metal. After dispersing the suspension for several minutes, the reaction system was evacuated to remove the air. A flow of cooling water was used to keep the suspension at approximately 284 K. The evolved H_2 was analyzed by gas chromatography (Shimadzu GC-8A with a thermal conductivity detector, 5 Å molecular sieve columns and Ar carrier gas).

1.3 Apparent quantum yield (AQY) measurement

The AQY of H_2 production was measured using the same aforementioned experimental setup but with a 420 nm band-pass filter. It was assumed that all incident photons were absorbed by the photocatalysts. The number of incident photons was measured using a grating spectroradiometer (EKO Instruments Co., Ltd., LS-100), and the AQY was calculated according to the following equation:

$$AQY(\%) = [2 \times n(H_2)] / n(\text{photons}) \times 100$$

where $n(H_2)$ and n(photons) indicate the number of generated H_2 molecules and the number of incident photons, respectively. The coefficient of 2 denotes that two photons are used to form one H_2 molecule.

1.4 Characterization

The morphology and particle size of the selenides were assessed by scanning electron microscopy (denoted as SEM, SU8020, Hitachi). X-ray diffraction (XRD) patterns were obtained using a Rigaku MiniFlex 300 powder diffractometer. UV-vis diffuse reflectance spectra (DRS) were recorded with a spectrophotometer (V-670, JASCO) equipped with an integrating sphere, with a Spectralon standard as a reference for baseline correction. Cross-sectional cuts of the particles were prepared by Ar ion milling using an ion slicer (EM-09100IS, JEOL), and then the amorphous damage layer on transmission electron microscopy (TEM) specimens produced by the

ion beam was removed by using a NanoMill Model 1040 (Fichione Instruments). A JEM-2800 system (JEOL) was used to record the scanning transmission electron microscopy (STEM), high-resolution transmission electron microscopy (HRTEM) images and selected area electron diffraction (SAED) patterns. Scanning transmission electron microscopy and energy-dispersive X-ray spectroscopy (STEM-EDS) characterization was carried out by using an X-MAX 100TLE SDD detector (Oxford Instruments). The binding energies were determined by X-ray photoelectron spectroscopy (XPS, PHI Quantera II spectrometer with an Mg Kα X-ray source), and normalized to the Au $4f_{7/2}$ (84.0 eV) for each sample. Reaction solutions were analysed by inductively coupled plasma-atomic emission spectroscopy (ICP-AES; ICPS-8100, Shimadzu).

Microsecond transient absorption measurements were performed using Nd:YAG laser system (Continuum, Surelite I; duration: 6 ns) with custom-built spectrometers.[3,4] Briefly, the IR probe light emitted from the $MoSi_2$ coil was focused on the sample and then the transmitted light was introduced to a grating spectrometer, which allows measurement of probe energies from 6000 cm^{-1} (1667 nm, 0.74 eV) to 1000 cm^{-1} (10 μm, 0.12 eV). The monochromated light was detected by a mercury cadmium telluride (MCT) detector (Kolmar), and the output electric signal was amplified with an AC-coupled amplifier (Stanford Research Systems, SR560, 1 MHz). 480 nm laser pulses (3 mJ $pulse^{-1}$) were used to excite the band gap of bare and cocatalysts-loaded ZnSe:CGSe photocatalysts. The time resolution of the spectrometer was limited to 1 μs by the response of the MCT detector. The output electric signal was amplified using AC-coupled amplifier with a bandwidth of 1 MHz, which can measure responses in the time scale of one microsecond to milliseconds. One thousand responses were accumulated to obtain the intensity trace at one wavenumber or decay curve. In order to rule out the thermal effects or IR emission, the absorption spectra and absorbance changes were determined after subtracting the measurements without probe light. The experiments were performed in vacuum at room temperature.

2. Results of Characterizations

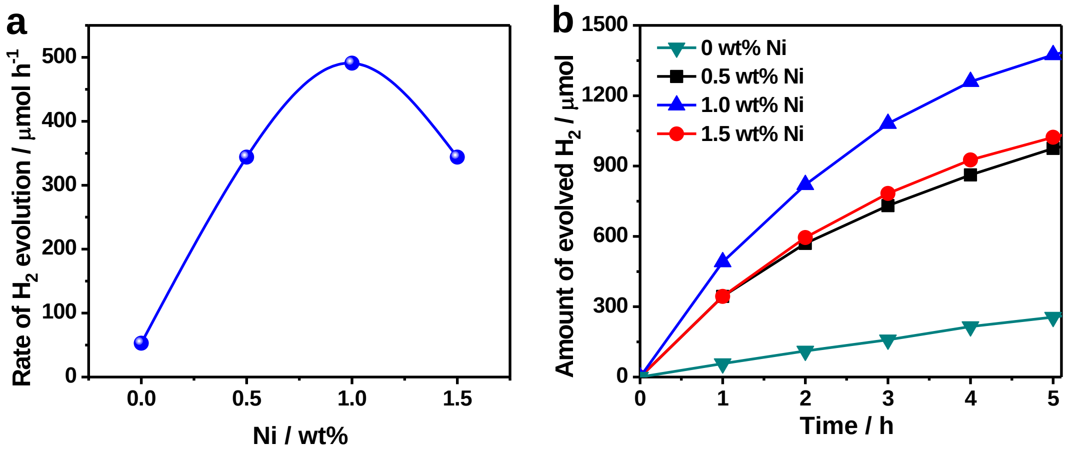

Fig. S1 Effect of loading amounts of Ni on the photocatalytic H_2 production: (a) H_2 evolution rates; (b) time courses of H_2 evolution. Experimental conditions: 0.15 g of photocatalyst, 0.1 mol L^{-1} Na_2S and 0.1 mol L^{-1} Na_2SO_3, 150 mL H_2O, 300 W Xe light, $\lambda > 420$ nm.

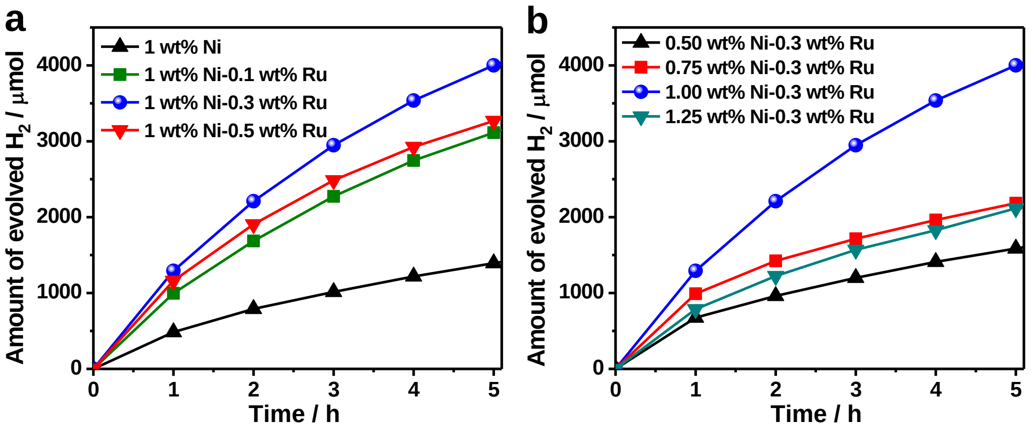

Fig. S2 Time courses of H_2 evolution for ZnSe:CGSe with different loading amounts of Ni and Ru: (a) different loading amount of Ru together with 1.0 *wt*% Ni; (b) different loading amount of Ni together with 0.3 *wt*% Ru. Experimental conditions: 0.15 g of photocatalyst, 0.1 mol L^{-1} Na_2S and 0.1 mol L^{-1} Na_2SO_3, 150 mL H_2O, 300 W Xe light, $\lambda > 420$ nm.

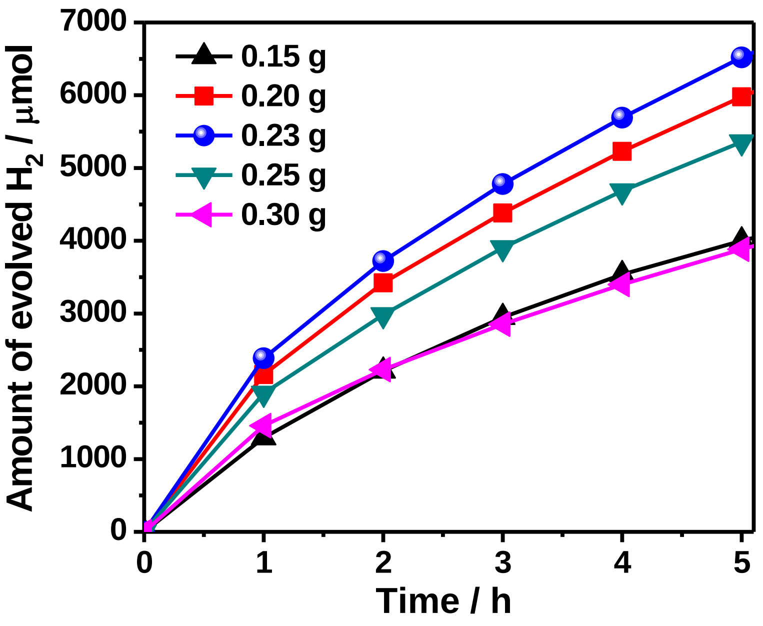

Fig. S3 Time courses of H_2 evolution for 1.0 *wt*% Ni-0.3 *wt*% Ru/ZnSe:CGSe photocatalyst with different introduced amounts. Experimental conditions: 0.5 mol L^{-1} Na_2S and 0.5 mol L^{-1} Na_2SO_3, 150 mL H_2O, 300 W Xe light, $\lambda > 420$ nm.

Table S1. Information of the state-of-the-art hydrogen evolution photocatalysts with different absorption edges.

Type	Semiconductor (available wavelength)	Co-catalyst	AQY	Refs
400 nm-class	$Cd_{0.5}Zn_{0.5}S$ (≤ 485 nm)	NiS	94% (at 425 nm)	5
500 nm-class	CdS (≤ 540 nm)	Pt-PdS	93% (at 420 nm)	6
600 nm-class	CdSe (≤ 650 nm)	Pt-PdS	45% (at 420 nm)	7
700 nm-class	ZnSe:CGSe (≤ 725 nm)	Ni-Ru	13.7% (at 420 nm)	This work

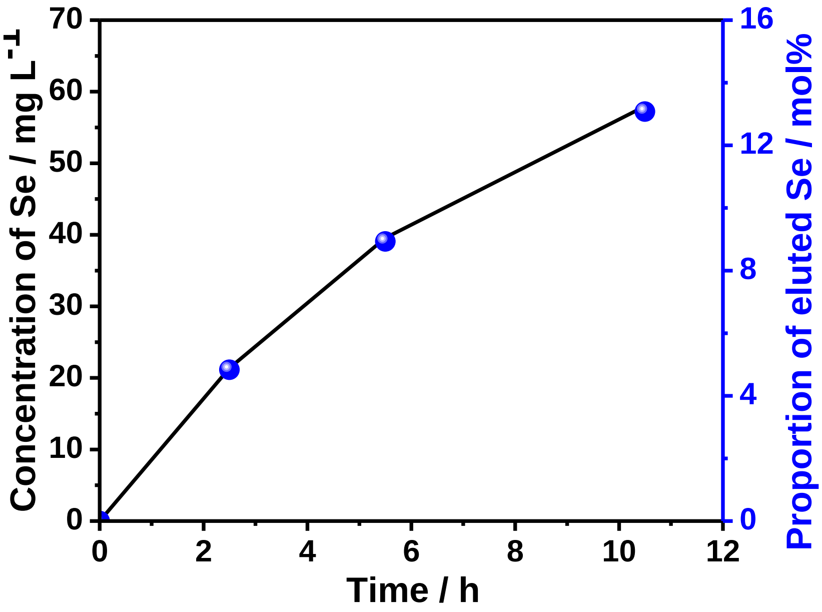

Fig. S4 Time course of the concentration of the dissolved Se element from 1.0 *wt*% Ni-0.3 *wt*% Ru/ZnSe:CGSe photocatalyst in the reaction solution. Experimental conditions: 0.5 mol L^{-1} Na_2S and 0.5 mol L^{-1} Na_2SO_3, 150 mL H_2O, 300 W Xe light, $\lambda >$ 420 nm.

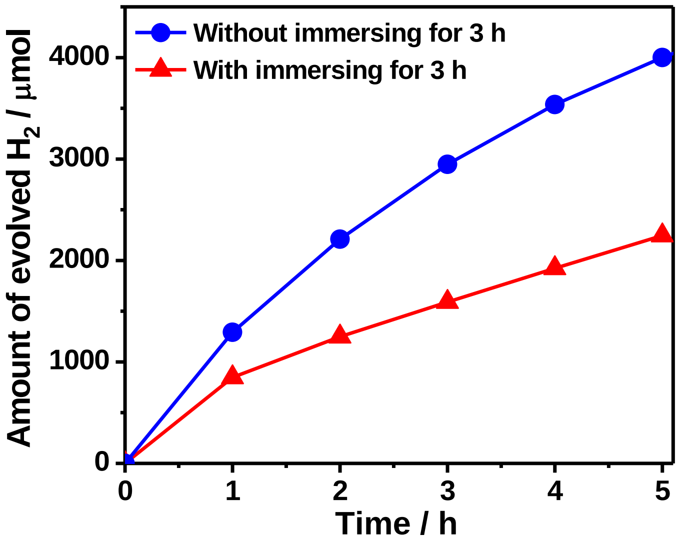

Fig. S5 Time courses of H_2 evolution for 1.0 *wt*% Ni-0.3 *wt*% Ru/ZnSe:CGSe photocatalyst with or without immersing in the reaction solution for 3 h under darkness. Experimental conditions: 0.5 mol L^{-1} Na_2S and 0.5 mol L^{-1} Na_2SO_3, 150 mL H_2O, 300 W Xe light, $\lambda > 420$ nm.

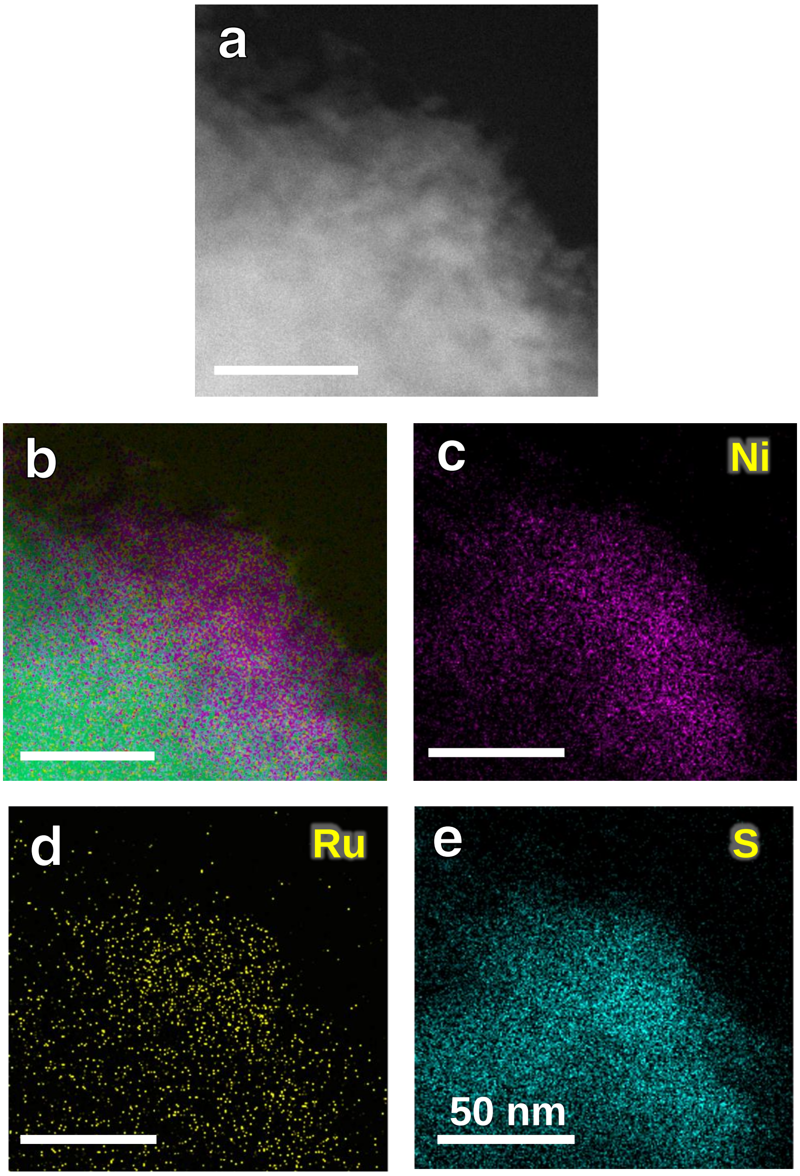

Fig. S6 STEM-EDS elemental mapping images of 0.5 *wt*% Ni-0.6 *wt*% Ru/ZnSe:CGSe sample: (a) STEM image; (b–e) STEM-EDS mapping images showing (b) a superimposition of all elements, (c) Ni, (d) Ru and (e) S. Herein, to make an obvious comparison of the locations of Ni and Ru elements, their loading proportions were adjusted to 0.5 *wt*% and 0.6 *wt*%, respectively.

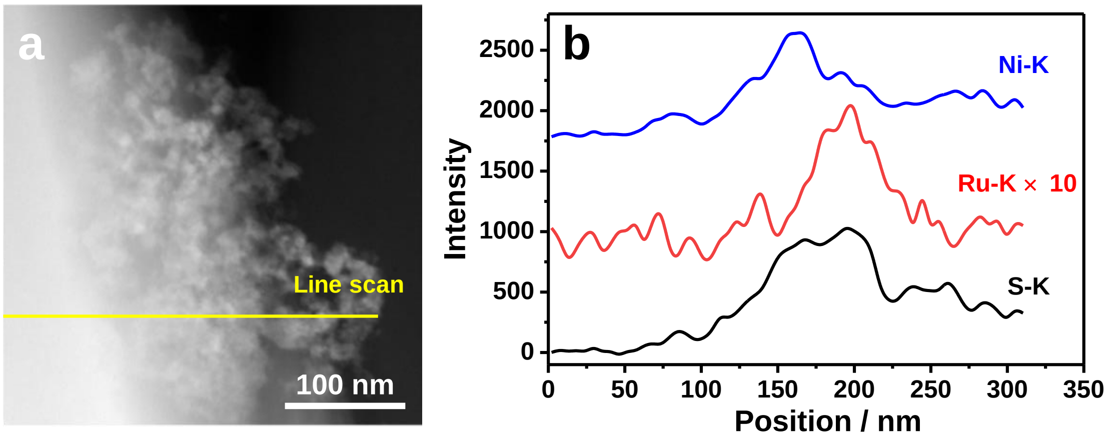

Fig. S7 STEM-EDS line analysis of 1.0 *wt*% Ni-0.3 *wt*% Ru/ZnSe:CGSe sample.

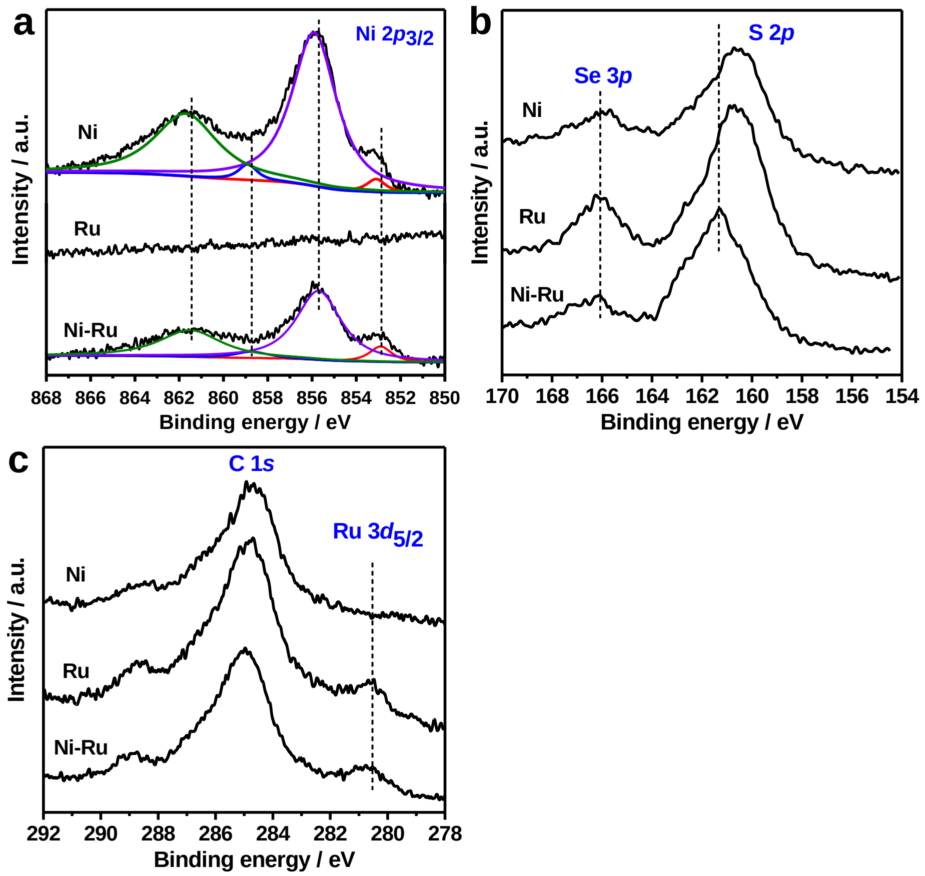

Fig. S8 XPS results of 1.0 *wt*% Ni/ZnSe:CGSe, 0.3 *wt*% Ru/ZnSe:CGSe and 1.0 *wt*% Ni-0.3 *wt*% Ru/ZnSe:CGSe samples. Herein, samples of 1.0 *wt*% Ni/ZnSe:CGSe, 0.3 *wt*% Ru/ZnSe:CGSe and 1.0 *wt*% Ni-0.3 *wt*% Ru/ZnSe:CGSe are denoted as Ni, Ru, and Ni-Ru for short in the above figures.

References:

1 Y. Goto, T. Minegishi, Y. Kageshima, T. Higashi, H. Kaneko, Y. Kuang, M. Nakabayashi, N. Shibata, H. Ishihara, T. Hayashi, A. Kudo, T. Yamada and K. Domen, *J. Mater. Chem. A*, 2017, **5**, 21242–21248.

2 S. Nandy, T. Hisatomi, G. Ma, T. Minegishi, M. Katayama and K. Domen, *J. Mater. Chem. A*, 2017, **5**, 6106–6112.

3 A. Yamakata, J. Vequizo and M. Kawaguchi, *J. Phys. Chem. C*, 2015, **119**, 1880–1885.

4 J. Vequizo, H. Matsunaga, T. Ishiku, S. Kamimura, T. Ohno and A. Yamakata, *ACS Catal.*, 2017, **7**, 2644–2651.

5 M. Liu, Y. Chen, J. Su, J. Shi, X. Wang and L. Guo, *Nat. Energy*, 2016, **1**, 16151.

6 H. Yan, J. Yang, G. Ma, G. Wu, X. Zong, Z. Lei, J. Shi and C. Li, *J. Catal.*, 2009, **266**, 165–168.

7 K. Khan, X. Tao, Y. Zhao, B. Zeng, M. Shi, N. Ta, J. Li, X. Jin, R. Li and C. Li, *J. Mater. Chem. A*, 2019, **7**, 15607–15614.
